# Malaria Research for Tailored Control and Elimination Strategies in the Greater Mekong Subregion

**DOI:** 10.4269/ajtmh.21-1268

**Published:** 2022-10-13

**Authors:** Jetsumon Sattabongkot, Liwang Cui, Sirasate Bantuchai, Sadudee Chotirat, Jaranit Kaewkungwal, Amnat Khamsiriwatchara, Kirakorn Kiattibutr, Myat Phone Kyaw, Saranath Lawpoolsri, Nay Yi Yi Linn, Lynette Menezes, Jun Miao, Wang Nguitragool, Daniel Parker, Pathomporn Prikchoo, Wanlapa Roobsoong, Patiwat Sa-angchai, Yudthana Samung, Jeeraphat Sirichaisinthop, Patchara Sriwichai, Kritsana Suk-uam, Suwich Thammapalo, Baomin Wang, Daibin Zhong

**Affiliations:** ^1^ Mahidol Vivax Research Unit, Faculty of Tropical Medicine, Mahidol University, Bangkok, Thailand;; ^2^ Department of Internal Medicine, Morsani College of Medicine, University of South Florida, Tampa, Florida;; ^3^ Department of Tropical Hygiene, Mahidol University, Bangkok, Thailand;; ^4^ Myanmar Health Network Organization, Yangon, Myanmar;; ^5^ Department of Public Health, Ministry of Health, Nay Pyi Taw, Myanmar;; ^6^ Department of Epidemiology, University of California at Irvine, Irvine, California;; ^7^ Office of Disease Prevention and Control 12, Ministry of Public Health, Songkla, Thailand;; ^8^ Department of Medical Entomology, Faculty of Tropical Medicine, Mahidol University, Bangkok, Thailand;; ^9^ Vector-Borne Disease Control Center, Department of Disease Control, Ministry of Public Health, Bangkok, Thailand;; ^10^ Vector Borne Disease Control Center 2.3, Ministry of Public Health, Tak, Thailand;; ^11^ College of Agriculture and Biotechnology, China Agricultural University, Beijing, China;; ^12^ Program in Public Health, University of California at Irvine, Irvine, California

## Abstract

The malaria landscape in the Greater Mekong Subregion has experienced drastic changes with the ramp-up of the control efforts, revealing formidable challenges that slowed down the progress toward malaria elimination. Problems such as border malaria and cross-border malaria introduction, multidrug resistance in *Plasmodium falciparum*, the persistence of *Plasmodium vivax*, the asymptomatic parasite reservoirs, and insecticide resistance in primary vectors require integrated strategies tailored for individual nations in the region. In recognition of these challenges and the need for research, the Southeast Asian International Center of Excellence for Malaria Research has established a network of researchers and stakeholders and conducted basic and translational research to identify existing and emerging problems and develop new countermeasures. The installation of a comprehensive disease and vector surveillance system at sentinel sites in border areas with the implementation of passive/active case detection and cross-sectional surveys allowed timely detection and management of malaria cases, provided updated knowledge for effective vector control measures, and facilitated the efficacy studies of antimalarials. Incorporating sensitive molecular diagnosis to expose the significance of asymptomatic parasite reservoirs for sustaining transmission helped establish the necessary evidence to guide targeted control to eliminate residual transmission. In addition, this program has developed point-of-care diagnostics to monitor the quality of artemisinin combination therapies, delivering the needed information to the drug regulatory authorities to take measures against falsified and substandard antimalarials. To accelerate malaria elimination, this program has actively engaged with stakeholders of all levels, fostered vertical and horizontal collaborations, and enabled the effective dissemination of research findings.

## INTRODUCTION

**Figure 1.**
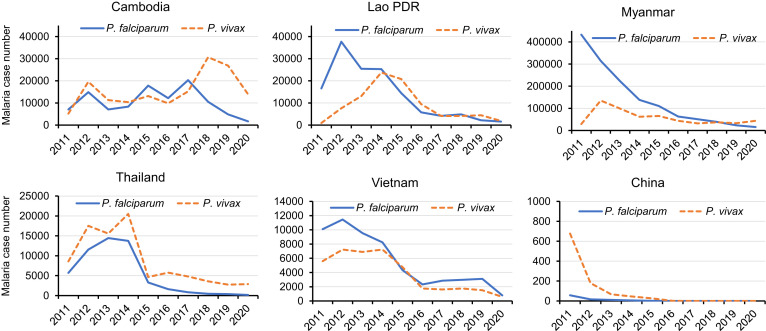
Annual cases of *P. falciparum* and *P. vivax* in the six GMS countries during 2011–2020. Data were extracted from the World Malaria Report 2021. This figure appears in color at www.ajtmh.org.

**Figure 2.**
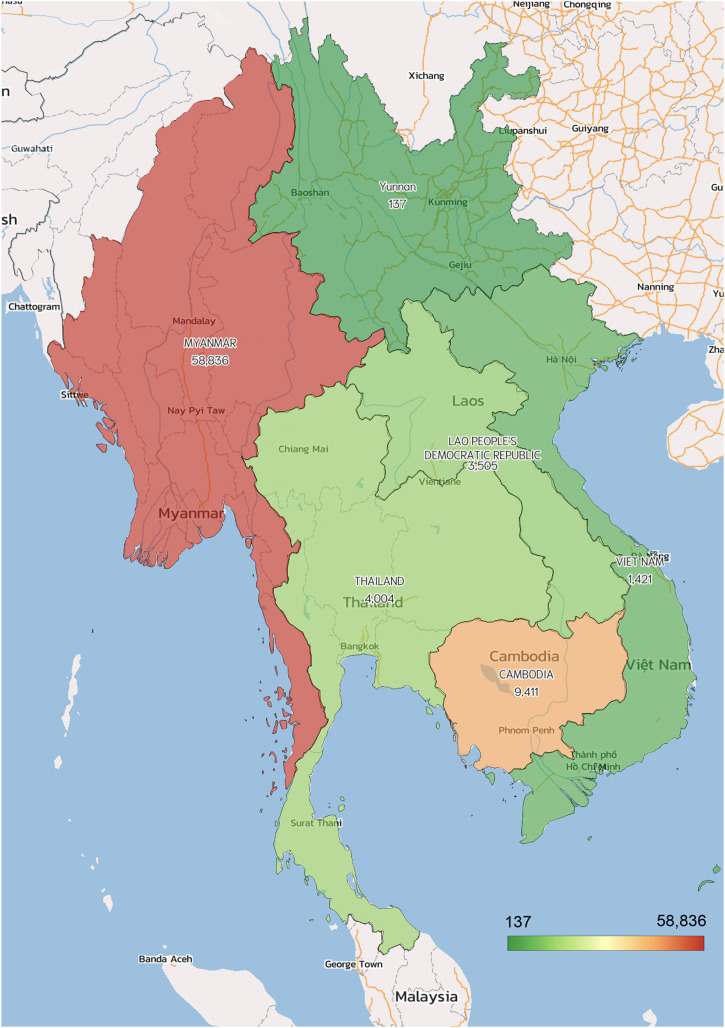
Distribution of confirmed malaria cases in the GMS in 2020. Note the number in China’s Yunnan Province indicates imported malaria cases. This figure appears in color at www.ajtmh.org.

Historically, malaria has been a serious public health problem in the Greater Mekong Subregion (GMS), hindering regional socioeconomic development.^1^^,^^2^ The GMS is comprised of Cambodia, China’s Yunnan and Guangxi provinces, Lao People’s Democratic Republic, Myanmar, Thailand, and Vietnam, which have tropical and subtropical climates and ecologies that are conducive to vector-borne diseases. Motivated by the achievements in malaria control over the past two decades, the six nations in the GMS joined an alliance to aim for malaria elimination by 2030.^3^ The first step of this regional campaign is to eliminate *Plasmodium falciparum* by 2025—a goal of both urgency and higher feasibility. This urgency is promoted by the emergence of artemisinin (ART) resistance in *P. falciparum*, detected first in West Cambodia a decade ago, and the fear of its potential catastrophic spread to hyperendemic areas in Africa.^4^ The availability of tools to effectively manage *P. falciparum* cases and interrupt its transmission (diagnosis, treatment, and even mass drug administration [MDA]) makes *P. falciparum* elimination in the GMS technically more feasible. However, *Plasmodium vivax* is much more resilient to conventional control measures, and its elimination requires more species-specific tools. This is well reflected in the different trends of *P. falciparum* and *P. vivax* malaria incidence in each GMS nation in the past decade, despite the overall decline of malaria incidence (Figure 1). In most countries, the reduction of the vivax malaria incidence leveled off after 2015, whereas Cambodia experienced a huge resurgence of vivax malaria in 2018–2019. Although the certification of malaria elimination in China by the World Health Organization (WHO) in 2021 has instilled further confidence in the regional goal, the immense variation in the distribution of malaria incidence also speaks for the varying challenges encountered by different GMS countries (Figure 2). Especially, the disruption of malaria control efforts by the COVID-19 pandemics may reverse the decades of progress against malaria, preventing the GMS from reaching the final goal in the planned timeframe.^5^

Many malarious nations had reduced their malaria incidence to the verge of elimination during the Global Malaria Eradication Program (GMEP) launched by WHO in the 1950s and had seen its comeback and resurgence with calamitous consequences.^6^ Although the causes for the malaria resurgence are multifactorial, including the weakening of the national malaria control program (NMCP) due to financial and operational constraints and technical problems, such as insecticide resistance in vectors and drug resistance in the parasites, they have altogether demonstrated the challenges in reaching the final goal during the last stage of malaria elimination. The dependence of GMEP on the universally adopted strategy (DDT for vector control and chloroquine [CQ] for malaria therapy) without a technical or strategic contingency plan also signifies the need for basic, applied, and operational research on malaria. In light of such a need, the Southeast Asian International Center of Excellence for Malaria Research (SEA-ICEMR) was established in 2010, aiming to build a collaborative research network within which academic institutions and malaria control stakeholders collaborate to investigate the existing and emerging problems in malaria control, identify solutions, develop and implement the new tools, and gather experience from the malaria elimination campaign.

## MALARIA EPIDEMIOLOGY AND SURVEILLANCE SYSTEM

Accurate and timely knowledge of malaria incidence is a prerequisite for delivering effective control efforts. A national malaria reporting system has been deemed essential at the final stage of malaria elimination in China, allowing real-time detection of malaria cases and implementation of the 1-3-7 strategy. This strategy stipulates case reporting within 1 day of case detection, case investigation within 3 days to identify the source of infection, and foci investigation and response including reactive case detection (RCD) and vector control within 7 days.

The Thai national electronic malaria information system (eMIS) has played a critical role in case detection, timely notification of the NMCP staff, and targeted application of control measures.^7^ The SEA-ICEMR staff has been actively involved in the development, maintenance, and improvement of this system. The eMIS allows local malaria centers to send individual malaria data directly to the centralized database^8^ while the data visualization platform assists prompt decision-making for national-level policy-makers and local control practitioners. For example, the eMIS allowed easy extraction and visualization of village-level malaria incidences in previous years and selection of high vivax incidence villages in Yala and Narathiwas Provinces to implement a pilot project of primaquine (PQ) MDA in 2019. We also used the eMIS to browse 10-year malaria incidence data to identify the hotspot malaria villages on the Thailand–Myanmar border to study how population movement influences malaria transmission using a mobile tracking application.

To better monitor the progress in malaria control, the SEA-ICEMR program has installed an integrated malaria surveillance system, incorporating cross-sectional surveys, active case detection, and passive case detection (PCD), to actively follow the malaria epidemiology in multiple sentinel sites along the international borders. Some sites were also malaria transmission hot spots located at or near settlements for internally displaced people or refugee camps.^9^^–^^11^ In contrast to the aggregated data usually collected by the national malaria databases, data collected from the ICEMR projects are based at the individual level. Case reports on the personal level have been used to identify high-risk populations and hotspot areas. Data generated from the program have been regularly shared with the local malaria control centers to identify urgent problems such as outbreaks and fine-tune their targeted control strategy. At the China–Myanmar border, our surveillance data have guided the local health bureaus to implement intensified vector control to suppress vivax malaria outbreaks.^11^ At the Tha Song Yang site in Western Thailand, we conducted malaria surveillance in a cohort of ∼4,000 people over 10 years through PCD of clinical cases and periodic cross-sectional surveys to determine infection prevalence.^9^ Although we identified a continuous reduction in the malaria incidence rate, we also detected the persistence of asymptomatic infections, which represented the majority of all malaria infections. Importantly, we demonstrated that asymptomatic carriers, particularly of *P. vivax*, are ubiquitously gametocytemic,^12^ and some can transmit the parasite to mosquitoes.^13^^,^^14^ The confirmation of the asymptomatic carriers as a critical reservoir of transmission helped lay the foundation for adopting the practice of treating asymptomatic individuals in Thailand. In addition, the current treatment regimen for falciparum malaria includes a low dose of PQ to interrupt transmission.

## DETECTION AND ELIMINATION OF RESIDUAL TRANSMISSION

### Incorporating molecular detection in malaria surveillance.

Early detection and prompt treatment are one pillar strategy for malaria control. As malaria incidence decreases, asymptomatic carriers have become critical parasite reservoirs sustaining malaria transmission and must be identified and treated for ultimate malaria elimination. They are invisible to the standard PCD-based surveillance for patients with acute malaria. They also have parasitemia (< 15 parasites/µL) below the limits of detection of a light microscope and rapid diagnostic tests,^15^^–^^18^ thus, requiring molecular approaches for detection. We have demonstrated the superior sensitivity of the molecular assays for malaria surveillance^19^^–^^23^ and definitive diagnosis of rare, often misidentified, malaria parasite species.^24^^–^^26^ These technologies were well-received and appreciated by the ministry and regional control authorities. In collaboration with the SEA-ICEMR, the Office of Disease Prevention and Control in Songkhla Province, Thailand, has recently initiated a project to use qPCR to detect malaria infection in transmission hotspots near the Thailand–Malaysia border. A central molecular diagnostic laboratory may provide the prevalence data from sentinel hotspots to guide targeted MDA.

### Implementing reactive case detection (RCD).

One of the strategies the GMS nations adopted to eliminate residual malaria transmission is the 1-3-7 strategy piloted in China.^27^ However, the implementation of this approach by different tiers of the NMCP teams varied among the GMS nations.^28^^,^^29^ RCD aims to actively detect additional infections, presumably asymptomatic, within about 10 households around the index case. Since the effectiveness of asymptomatic case detection depends largely on the sensitivity of diagnostic methods, incorporating molecular diagnosis for the RCD cases is currently under consideration by our collaborating regional authorities.

## CROSS-BORDER MALARIA INTRODUCTION

In the GMS, malaria transmission between the two sides of the border can be radically different.^30^ Extensive population movement at the borders favors malaria transmission and cross-border introduction. Our epidemiology and population genetic studies at the China–Myanmar border have provided solid evidence for malaria importation in the border counties of China,^31^^–^^34^ motivating the enactment of strengthened border surveillance and intensified prevention efforts in the buffer zone. Similarly, cross-border population movement is a significant factor for the persistence of malaria transmission along the Thailand–Myanmar border.^35^^–^^38^ Our studies in China and Thailand identified that *P. falciparum* was mainly associated with cross-border migration activities, whereas *P. vivax* was locally transmitted.^31^^,^^38^^,^^39^ In the GMS, forest-goers carry a significantly higher risk of malaria infection and are targeted for malaria prevention.^40^ Work conducted in a Thailand–Myanmar border area showed that only 9% of forest-goers used ITN/LLIN when they went to the forest.^41^ Together, these findings indicate the vulnerability of malaria reintroduction from neighboring countries due to cross-border activities. They provide strong evidence for the NMCPs to strengthen the cross-border malaria surveillance system to monitor and rapidly respond to introduced cases.

In 2020, Myanmar accounted for ∼70% of the regional malaria burden,^42^ and the slight increase in malaria incidence over 2019 was due to vivax malaria outbreaks in its eastern border townships. Although a 3% decrease in malaria cases was recorded in 2021 compared with 2020, there was a 60% decline in the number of suspected cases tested. Although the waves of the COVID-19 pandemic disrupted malaria service delivery, the political crisis that emerged after February 2021 has further devastated the public health system, resulting in a shortage of health staff and a reduction in the coverage of malaria control activities.^43^ Travel restrictions and heightened security concerns hindered the timeliness and accuracy of case reporting, affected the delivery of health services to high-risk populations, and delayed responses to malaria outbreaks. The NMCP currently collaborates with WHO and various stakeholders to address the border malaria issue using a decentralized approach emphasizing community-based health workers. As an imminent threat of cross-border malaria introduction into neighboring countries, malaria in Myanmar desires enhanced international attention and strengthened control efforts, especially for high-risk groups like the internally displaced people, at this difficult time.

## VECTOR SURVEILLANCE AND INSECTICIDE RESISTANCE

As an essential tool to achieve malaria elimination, vector control relies on the knowledge of vector biology and ecology. One research activity of the SEA-ICEMR is to conduct vector surveillance at the malaria hotspots to provide updated information on vector species composition, seasonal dynamics, biting behaviors, and insecticide resistance.^44^ We identified areas within 1 km from the forest edge as the high-transmission zones, where *Anopheles maculatus* is the dominant species, followed by *An. minimus* and *An. dirus*.^45^ We identified changes in the biting behavior of primary vectors, indicating that human populations were experiencing more prolonged exposure to mosquito bites during the early evening (18:00–21:00 hour) and the early morning (05:00–06:00 hour).^46^ Ecological changes due to human agricultural activities such as deforestation have resulted in rapidly changing vector species composition, with some new malaria vectors playing more important roles in malaria transmission.^39^ The updated vector information has been used to build a spatial mathematical simulation model to quantitatively evaluate the impact of interventions on interrupting malaria transmission in the border villages. The information gathered on local vectors has helped guide the vector control staff to determine the time and frequency of vector control practice, such as the indoor residual spraying. Our team has also been keenly following the sensitivity of primary malaria vectors to insecticides currently used in vector control. The emerging pyrethroid resistance discovered at the Thailand–Myanmar border provides the knowledge base for adjusting the insecticide use policy.

## ARTEMISININ RESISTANCE IN FALCIPARUM MALARIA

The emergence of resistance to ART in *P. falciparum* in Cambodia a decade ago prompted extensive research and surveillance activities in the GMS. The current epicenter of ART resistance is in the Eastern GMS, including Cambodia, Vietnam, Laos, and Eastern Thailand.^47^^,^^48^ Together with resistance to the partner drugs piperaquine and mefloquine, ART resistance resulted in high failure rates of two ART-based combination therapies (ACTs), dihydroartemisinin-piperaquine and artesunate-mefloquine, in the Eastern GMS.^49^ To mitigate this problem, triple ACTs, combining an ART derivative with two partner drugs that exert counterbalancing resistance selection, have been evaluated.^50^ Our in vivo and in vitro monitoring of *P. falciparum* resistance to ART and partner drugs showed high clinical efficacies of ACTs and a general lack of resistance to piperaquine and mefloquine in Myanmar.^51^^–^^53^ Given Myanmar’s geographical location in bridging South and Southeast Asia, an important goal of the NMCP is to restrict the spread of ART-resistant parasites to central and Western Myanmar. Although the trends of falciparum malaria in the GMS nations are encouraging (Figure 1), it remains a challenge to eliminate falciparum malaria by 2025 amid the COVID-19 pandemics and the political turmoil in Myanmar.

## EFFECTIVE DRUGS TO ELIMINATE VIVAX MALARIA

*Plasmodium vivax* has become the predominant parasite in most endemic areas of the GMS.^54^ We reasoned that the increased proportion of vivax malaria might be partially attributed to the reduced efficacy of the frontline schizonticide CQ. Thus, we have been conducting longitudinal CQ efficacy studies in sentinel sites at the East and West borders of Myanmar.^55^^–^^57^ These studies have documented deterioration of the clinical efficacy of CQ in several Eastern areas of Myanmar. The most recent study in Northeastern Myanmar showed cases, where CQ failed to clear *P. vivax* parasitemia within 7 days, suggesting high-grade resistance.^56^ We also used an ex vivo assay to profile drug susceptibilities in clinical vivax isolates, suggesting the emergence of CQ resistance in the *P. vivax* populations along the Eastern border of Myanmar.^58^^,^^59^ The presence of mixed *P. vivax/P. falciparum* infections and difficulties in correctly diagnosing all malaria species have promoted the adoption of ACT as a unified malaria treatment in this region. Cambodia adopted a unified ACT policy for treating all malaria in 2012. While all ACTs should be highly efficacious as schizonticides to treat blood-stage *P. vivax* infections, only some (e.g., dihydroartemisinin-piperaquine, artemether-lumefantrine) have been vigorously evaluated for their compatibility with PQ.^60^^,^^61^

The resilience of vivax malaria to conventional malaria control measures is due primarily to its ability to form dormant hypnozoites that are activated later to cause malaria relapses. PQ is the only approved hypnozoiticidal drug for the radical cure of vivax malaria. The GMS countries use the WHO-recommended PQ regimen (0.25 mg/kg/day for 14 days), but it is under-prescribed in endemic populations due to the danger of severe hemolysis PQ may cause in patients with glucose-6-phosphate dehydrogenase deficiency (G6PDd). In addition, this long treatment regimen is associated with inherent poor compliance. Yet, PQ MDA during the winter break has been instrumental in eliminating temperate-zone vivax malaria from central China,^62^ where the risk of PQ-induced acute hemolytic anemia is minimal. However, its feasibility for vivax malaria elimination in the endemic settings of the GMS, where G6PDd is often prevalent in the human populations, remains to be tested. In collaboration with the Ministries of Health (MOH) of Thailand and Myanmar, we have recently conducted a cluster-randomized crossover trial as a proof-of-concept study to evaluate the effectiveness and operational feasibility of PQ MDA. Overall, the study revealed a reduction in *P. vivax* incidence in the intervention arm, but it was not significantly different from that of the control arm. Nonetheless, this study proved the feasibility of G6PD test-based PQ MDA, whereas a conclusive demonstration of its effectiveness for vivax malaria elimination requires further evaluation in a larger population. In addition, this study also generated several interesting findings. We detected many asymptomatic cases in the study populations not captured by the routine PCD-based malaria surveillance system, further justifying future MDA-based preventive measures. This study also obtained new information about the prevalence and distribution of G6PDd in the study populations, which differed from those previously reported from other sites in the two countries. The health authorities at the ministerial and local levels have recognized the values of this pilot study and took subsequent actions based on the evidence-based statistics from this work.

## ANTIMALARIAL DRUG QUALITY SURVEYS

Another predicament for malaria elimination in the GMS is the circulation of falsified antimalarial drugs, especially ACTs.^63^ To enable the point-of-care quality control of antimalarial drugs under remote endemic settings, we developed lateral flow dipsticks, similar to the malaria RDTs, for rapid assessment of ACT qualities.^64^^–^^67^ In light of the historical circulation of fake artesunate drugs in Myanmar,^68^ we performed a follow-up assessment of ACT drugs in private sectors of Myanmar.^69^ Our survey revealed an astonishingly high proportion of ART monotherapies (>35%) and identified an artesunate injection sample as a falsified drug containing no active ingredient. This finding has been communicated promptly with the regulatory authorities in Myanmar, which paid close attention to the problem and implemented corrective actions.

## COMMUNITY ADVOCACY AND ENGAGEMENT

Our SEA-ICEMR has actively engaged with stakeholders of all levels—ministerial officials, regional malaria control health officers, and community participants. Especially for the PQ MDA trial, community health workers have been the key performers in drug delivery and follow-up activities. Community involvement has provided us the opportunity to explore the social aspects of malaria. Our recent survey on bed net usage in malaria hotspot villages of the Thailand–Myanmar border revealed that only one-third of the households reached the standard level of one ITN/LLIN per two persons specified by the MOH.^41^ Social science studies were also performed to determine the acceptance of new malaria control initiatives such as the PQ MDA. The project activities to evaluate a health initiative’s operational feasibility have fostered vertical and horizontal collaborations among all stakeholders and strengthened the relationships among the villagers, healthcare providers, and village health volunteers. Through this study, we found that most community members and healthcare providers in Myanmar showed enthusiasm toward and high acceptability of the targeted PQ MDA. Among them, respondents with secondary education level who had prior malaria experience and considered that malaria elimination could be achieved in their villages were most likely to show a willingness to participate. The results stressed the need for community engagement and health education before organizing the treatment program to bolster the community buy-in.^70^

## CONCLUSION

The SEA-ICEMR has been conducting collaborative multidisciplinary research on malaria parasites, vectors, and human populations, aiming to identify existing and emerging problems and develop relevant solutions to expedite malaria elimination in the GMS. The active engagement with stakeholders at all levels has enabled the effective dissemination of findings from the translational research activities. The ICEMR project also conducted training of next-generation malariologists and entomologists, which will have far-reaching impacts on sustaining malaria research to reach the ultimate goal of regional malaria elimination. Evidence collected from the past research identifies the following elimination strategies: 1) strengthen the surveillance system to ensure timely case reports and responses using the 1-3-7 approach; 2) develop programs targeting high-risk populations such as the forest-goers to eliminate residual transmission; 3) implement targeted MDA strategy to eliminate malaria in the hotspot areas; 4) improve the implementation of the radical cure of vivax malaria; 5) install national drug quality surveillance systems to ensure effective malaria case management; 6) effectively engage community participation in the elimination efforts; and 7) promote international collaboration to reduce and prevent cross-border parasite introduction.
